# Neurocognitive Markers During Prolonged Breath-Holding in Freedivers: An Event-Related EEG Study

**DOI:** 10.3389/fphys.2019.00069

**Published:** 2019-02-06

**Authors:** Fabian Steinberg, Michael Doppelmayr

**Affiliations:** ^1^Department of Sport Psychology, Institute of Sport Science, Johannes Gutenberg-University Mainz, Mainz, Germany; ^2^Centre of Cognitive Neuroscience, University of Salzburg, Salzburg, Austria

**Keywords:** hypoxemia, hypercapnia, ERP, electroencephalography, apnoea diving, P300, VEP

## Abstract

Since little is known concerning the psychological, cognitive, and neurophysiological factors that are involved in and important for phases of prolonged breath-holding (pBH) in freedivers, the present study uses electroencephalography (EEG) to investigate event-related neurocognitive markers during pBH of experienced freedivers that regularly train pBH. The purpose was to determine whether the well-known neurophysiological modulations elicited by hypoxic and hypercapnic conditions can also be detected during pBH induced hypoxic hypercapnia. Ten experienced free-divers (all male, aged 35.10 ± 7.89 years) were asked to hold their breath twice for 4 min per instance. During the first pBH, a checker board reversal task was presented and in the second four-min pBH phase a classical visual oddball paradigm was performed. A visual evoked potential (VEP) as an index of early visual processing (i.e., latencies and amplitudes of N75, P100, and N145) and the latency and amplitude of a P300 component (visual oddball paradigm) as an index of cognitive processing were investigated. In a counter-balanced cross-over design, all tasks were once performed during normal breathing (B), and once during pBH. All components were then compared between an early pBH (0–2 min) and a later pBH stage (2–4 min) and with the same time phases without pBH (i.e., during normal breathing). Statistical analyses using analyses of variance (ANOVA) revealed that comparisons between B and pBH yielded no significant changes either in the amplitude and latency of the VEP or in the P300. This indicates that neurocognitive markers, whether in an early visual processing stream or at a later cognitive processing stage, were not affected by pBH in experienced free-divers.

## Introduction

Freediving is a specialized underwater activity which is being performed by more and more people, be it for competitive, or recreational purposes. Freediving frequently requires the human body to approach the individual limit in breath-holding (BH), necessitating special adapting mechanisms, including extreme responses. Maximal BH is not only determined by physiological but also by a series of psychological factors and thus is often described as a unique, psycho-physiological state (Schagatay, 2009; Laurino et al., 2012; Steinberg et al., 2017). However, despite the various psychological factors that determine maximal BH-time, the bulk of past and ongoing research focuses on the physiological aspects of the respiratory, circulatory, and metabolic processes (Ferretti, 2001; Bain et al., 2018b; Fitz-Clarke, 2018), with special emphasis either on fundamental cardiovascular dynamics and processes or on the pathophysiological aspects (Lindholm and Lundgren, 2009). Consequently, it is well known that as soon as humans stop breathing, a series of physiological reactions occur (Foster and Sheel, 2005; Lindholm and Lundgren, 2009; Fagoni et al., 2015; Sivieri et al., 2015; Eichhorn et al., 2016; Fitz-Clarke, 2018), and all of them are most probably involved in maintaining oxygen supply to sensitive regions such as the brain and the heart and also protecting against hypercapnia (Foster and Sheel, 2005; Joulia et al., 2009). Hypercapnia (i.e., increases in carbon dioxide levels) additionally elicits asphyxia sensation, which also induces additional physiological responses and adaptation mechanisms such as reduced ventilatory responses in breath-hold divers (Davis et al., 1987; Grassi et al., 1994; Foster and Sheel, 2005). Some of those physiological studies additionally draw attention specifically to the brain [e.g., cerebral blood flow (CBF)] and indicate that blood oxygen saturation decreases (i.e., hypoxia) and blood carbon dioxide increases (i.e., hypercapnia) over the course of prolonged BH (pBH), eventually also affecting the brain (Palada et al., 2007; Dujic et al., 2013; Willie et al., 2015; Bain et al., 2015, 2016, 2017, 2018a).

Due to this emphasis on the cardiovascular system, little is known regarding the unique mental state and associated electrical brain activity from a neurophysiological and cognitive-psychological perspective, even though it has been argued that there are several other aspects related to prolonged pBH that might be worth studying (Schagatay, 2009; Laurino et al., 2012; Steinberg et al., 2017). pBH requires cognitive top-down control to actively inhibit the increasing drive of the respiratory system due to air hunger, maintain BH and focus on the task goal (i.e., motivation) by simultaneously staying maximally relaxed or move as efficiently as possible (i.e., limiting energy consumption). Some of these functions are interrelated with physiological mechanisms such as the interoceptive awareness of homeostatic changes (Liotti et al., 2001; Craig, 2002; Steinberg et al., 2017). Thus, pBH performance up to the individual limit requires strong emotional self-regulatory components, which is a factor that is potentially reflected in a lateralised cortical processing (i.e., stronger left prefrontal activity at the end of a 4 min long pBH phase), as observed in our recent study using electroencephalographic (EEG) measurements (Steinberg et al., 2017). Except for a few other studies using EEG to measure general brain activity patterns and emotional responses (Menicucci et al., 2014; Ratmanova et al., 2016; Steinberg et al., 2017), almost nothing is known about how higher level cortical processing and neuro-electrical activity is affected by pBH, possibly due to the limitations of measuring such aspects within a short time frame during pBH phases.

However, neuro-electrical measurements in hypoxia, hypercapnia or hyperoxia have observed changes in the frequency spectra of the EEG or magnetoencephalography (MEG) (Meyer and Waltz, 1960; Kraaier et al., 1988; van der Worp et al., 1991; Ozaki et al., 1995; Bloch-Salisbury et al., 2000; Halpern et al., 2003; Papadelis et al., 2007; Tsarouchas et al., 2008; Hall et al., 2011; Thesen et al., 2012; Sheng et al., 2017), while it is thought that hypercapnia compared to hypoxia has a stronger effect on oscillatory activity (Wang et al., 2015). In EEG based event-related potential (ERP) studies, as an example, Tsarouchas et al. (2008) found modulations in the ERPs while performing visuo-cognitive tasks (go/no-go paradigm) of the EEG during moderate hypobaric hypoxia. Several other hypoxia studies show that early visual functions within the occipital lobe of the brain, especially, are sensitive to hypoxia (e.g., Kobrick, 1983; Fowler et al., 1993; Fowler and Nathoo, 1997), for example, as evidenced in already decreased visual evoked potential (VEP) amplitudes at high altitudes of 4300 m (Forster et al., 1975; Singh et al., 2004). It was also experimentally observed that a portion of the deficits in higher cognitive processing was due to such early visual impairments (Fowler et al., 1993; Fowler and Nathoo, 1997). One ERP study in humans breathing elevated CO_2_ concentration revealed no effects on N1, P2, or P3 in an auditory signal-detection task in moderate hypercapnia and this did not cause any change in cognitive function (Bloch-Salisbury et al., 2000). A more recent MEG based study with 5% CO_2_ breathing clearly found decreases in neural activity, as indicated by reduced ERP amplitudes and increased latencies in early visual and late cognitive processing streams (Thesen et al., 2012).

Here, we report an experiment to obtain brain-electrical neurocognitive markers, and more specifically ERP, by EEG during two pBH phases of 4 min in experienced free-divers while they perform a classical checker board task to elicit transient VEPs and a visual oddball paradigm to elicit the well-known P300 (i.e., a positive EEG deflection occurring around 300 ms after stimulus onset) component in the brain. VEP and P300 provide insights into early visual processing within the occipital lobe and higher cognitive processing in parietal and frontal brain areas (Kok, 2001; Odom et al., 2010). As both markers are sensitive to changes in blood gas compositions, here we questioned whether ERP modulations can be observed during phases of pBH in experienced freedivers compared to phases of normal breathing. Besides case reports (Schellart and Reits, 1999), and another ERP study on emotional task-evoked responses (Menicucci et al., 2014), such neurocognitive markers (ERPs) have never been systematically recorded during pBH phases even though they might provide new insights about perceptual and central cognitive processing during the unique psycho-physiological state of pBH. Using such a task-evoked activity approach and measuring changes in the brain dynamics at a high temporal resolution might also allow the detection of even subtle changes in electrophysiological mechanisms evoked by pBH induced hypoxic hypercapnia (Tsarouchas et al., 2008).

## Materials and Methods

### Participants

Ten right-handed, healthy and well-trained male freedivers (35.10 ± 7.89 years) participated in this study. Freedivers were only included in this study if they were able to hold their breath for at least 5 min. Maximum static BH duration ranged between 317 and 451 s, with a mean of 368.8 ± 39.87 s, and self-stated apnoea training frequency ranged between two to five times per week. Prior to the experiment, all participants were informed of the purpose of the study and signed an informed consent form. The test protocol followed the Helsinki declaration and was approved by the ethics committee of the Deutsche Gesellschaft für Psychologie.

### EEG, Heart Rate and Arterial Oxygen Saturation Measurements

Electroencephalography was recorded (BrainVision Recorder 1.2 Brain Products, Germany) at a sampling rate of 1000 Hz (notch filter at 50 Hz) by a portable actiCAP system (Brain Products) equipped with 32 active Ag/AgCl electrodes and caps that were adapted to individual head size. EEG data was recorded according to the international 10:10 system at electrode sites Fp1, Fp2, F7, F3, Fz, F4, F8, FC5, FC1, FC2, FC6, T7, C3, Cz, C4, T8, CP5, CP1, CP2, CP6, P7, P3, Pz, P4, O1, Oz, and O2. Vertical and horizontal electrooculograms (EOG) were measured by two additional electrodes beside and above the right eye to detect eye movements. The reference electrode was placed at the nose tip and the ground electrode at AFz. A1 and A2 electrodes at the ears were used for subsequent re-referencing during offline processing. Impedance was maintained below 5 kOhm by using SuperVisc^TM^ electrode gel filled in each electrode (EASYCAP GmbH, Germany) for conductivity. A digitalized pulse oximeter (CMS60D, Contec, China) positioned on the left index finger was used to monitor arterial oxygen saturation (SpO_2_) and heart rate (HR).

### Experimental Tasks: Visual Oddball and Checker Board Reversal Task

Due to the evidence that even mild hypoxia and hypercapnia can be detected by neuro-electrical measures and are sensitive to hypoxic and hypercapnia states (cf. Introduction), we investigated whether this is the case for pBH (i.e., 4 min), and to this end, we selected freedivers as participants that were capable of at least 5 min of maximum BH. They were asked to perform dry pBH tasks while performing a checker board task (i.e., eliciting a VEP) and an Oddball task (i.e., eliciting a P300). Due to the main measure being EEG, we explicitly did not measure individual maximal BH phases as this would result in EEG data with much noise due to movement artifacts coming from increases in involuntary breathing movements at the end of maximal BH. By dividing the BH phase into an early and a late BH phase, we investigated whether the unique psycho-physiological state provoked by the pBH time-dependent increases in psychological and physiological demands affects visual and cognitive processing. Additionally, we also exploratory questioned whether the expected short and mild hypoxic hypercapnia state (i.e., “only” 4-min of BH) at a prolonged phase of BH does sufficiently propagate to the visual system, in so far that it can be detected by changes in VEPs in the occipital lobe as measured by EEG. In order to also observe if such deficits (if any) in the visual system propagate to later cognitive processes or whether cognitive functions and later evoked potentials (i.e., P300) are affected independently of any visual system deficits, we let the same divers perform a second 4 min BH while they performed a visual oddball paradigm (i.e., eliciting the P300 component). We chose both these tasks because of the potential sensitivity to hypoxia and hypercapnia and due to the possibility of getting reliable and valid EEG data in a very short time frame with a comparable number of stimuli in each phase.

#### Oddball Task

**FIGURE 1:**
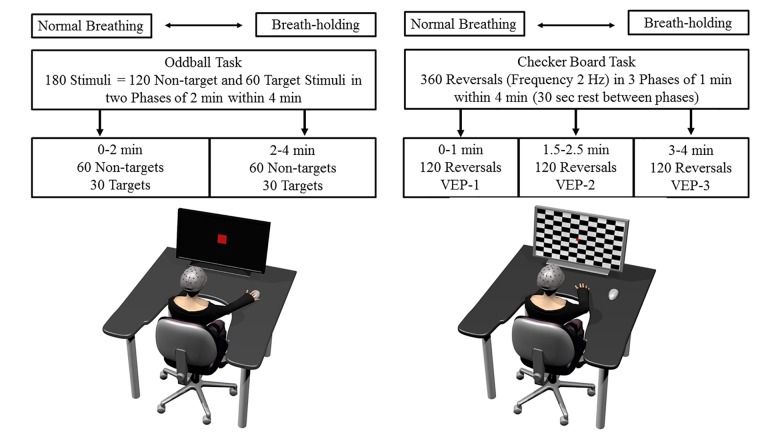
Schematic overview of the experimental tasks, setup, and procedure. The drawing on the left shows the participant performing the oddball task and the right side drawing the checker board task. More details regarding each task, procedure, and the order of tasks are provided in the text.

A visual oddball paradigm was applied. The task consists of presenting a series of repetitive stimuli of one shape along smaller number of deviant stimuli to which the participant must usually react fast and accurately by pressing a button. Those target stimuli (i.e., the deviant stimuli) elicit the prominent ERP P300, which is characterized as a peak in the amplitude following about 300 ms after stimulus onset, and represents a component frequently used in the past in order to analyze cognitive processing on a neurophysiological level (review in Kok, 2001). The latency of the P300 component has frequently been associated with task difficulty and timing of mental processes, and the amplitude is most often used as an index of processing intensity (Kok, 2001). The inherent task demands of the oddball task require active cognitive processes from a participant, and the elicited P300 waveform represents a kind of real-time marker for constantly updating working memory and focused attention (Iv et al., 2010). Thus, P300 gives valuable insight into cognitive processing efficiency and can serve as a neurobiological marker for pathophysiological mechanisms (Polich, 1998, 2004). The oddball task included a presentation on a computer screen of target stimuli (2 × 2 cm) to which the participants had to react as quickly as possible by pressing a button on the keyboard using the right index finger (Figure 1). Non-targets were red circles that were larger in size (3 × 3 cm) than the target squares and required no response. Each stimulus was presented for 500 ms and was preceded by a fixation cross and inter-stimulus interval between 1100 and 1500 ms. The entire task lasted 4 min and included 180 stimuli, of which 60 stimuli were targets and 120 non-targets (i.e., 33.3% probability), all presented randomly. Our subsequent, predefined ERP analysis separated each task into blocks of similar length by dividing the 4-min BH (or normal breathing period, see below) into a first, 2-min phase with no substantial decrease in blood oxygenation and a second phase (2–4 min) in which a decline in oxygen saturation was expected. To maintain a counterbalanced and constant volume of stimuli between these two phases, the algorithm of the software guaranteed that 90 stimuli (30 targets and 60 non-targets) were presented within the first 2 min and the other 90 stimuli (30 targets and 60 non-targets) were presented in the last 2 min.

#### Checker Board Task

Transient visually evoked potentials were elicited by a classical white and black checker board reversal task (Figure 1). This pattern-reversal VEPs as used in the present approach, include a clinically effective complex consisting of three peaks N1, P1, and N2 (deflections at about 75, 100 and 145 ms after pattern reversal). It is thought that the N1 and P1 are processed in striate brain areas, while N2 is thought to be located in the extrastriate visual cortex and thus is an index of functional visual processing and integrity (Seki et al., 1996; Brecelj et al., 2002). The checker (squared checkers) board task was presented on the screen which changed its pattern (i.e., from black to white squares and vice versa) at a rate of 2 Hz, so that two pattern reversals appeared in 1 s. A red fixation dot was presented in the middle of the screen at the corner of four checks. Following standard clinical recommendations, the eye-to-screen distance was 1000 mm and stimulus field size 48 min of arc (Odom et al., 2010). This kind of pattern reversal task elicits three typical deflections in the EEG signals at the occipital lobe. With latency variations depending on factors such as luminance and pattern contrast, a first negative deflection usually occurs around 75 ms (N75), a positive at 100 ms (P100) and a second negative at 145 ms (N145) (Odom et al., 2010). Three phases of the same checker board reversals, each lasting exactly 1 min (i.e., 120 checker reversals), were presented during the 4 min. The first 1-min phase started directly after the BH start, followed by 30 s rest; a second 1-min checker phase, again 30 s of rest, and finally a last 1-min checker phase until the end of BH. Rest phases were included in order to draw attention to the fixation point, since 4-min of constant checker reversals would be too demanding.

The “Presentation” (Neurobehavioral System^®^, United States) software was used, for stimulus programming and presentation. For synchronization of stimuli and EEG, TTL input signals were sent by the software, indicating the task start, the exact time of stimulus onset, kind of stimulus, end of the first 2 min and participant’s response onset (i.e., button press for the target stimuli in the oddball task). In the software code of the checker board task, the TTL signal to the EEG was sent at the reversal command. Due to the screen’s refreshing rate (i.e., the pattern reversal appeared one refreshed screen later), a constant time delay of 16–17 ms was included in the EEG signal, thus provoking a constant delay of VEPs latencies.

### Experimental Procedure

The first task in this experiment was for the participant to hold their breath for 4 min without any parallel task or visual stimuli on a screen and with eyes open. This resting pBH measure was used to analyze brain oscillatory modulations in the course of the BH compared to resting (no BH, i.e., normal breathing) condition performed with eyes open prior to BH. Data analysis of this task has already been presented in another publication and is therefore not reported here (Steinberg et al., 2017). However, the data presented here is based on the same 10 freedivers and followed another approach (brain oscillations) such as this data being presented in a separate publication. Since this task was performed before the oddball and checker board tasks, no significant carry-over effects are expected, except possible short-term effects in terms of BH capability.

A computer screen on a table was positioned 100 cm from the participants’ faces, while they were seated in a chair (Figure 1). They were asked to reduce body and head movements during all EEG measures. All participants were allowed to individually prepare (exhausting hyperventilation was restricted, no time limit) for the BH phase, during which no EEG registrations were made. In total, participants were requested to hold their breath three times for 4 min each, the first for resting measures (brain oscillations analysis, see Steinberg et al., 2017), the second for the oddball task and the third for the checker board task. Both the oddball and the checker tasks were performed once in BH and once under normal breathing (B), and the order was counterbalanced across participants. The start of each task was self-initiated by an acoustic signal (finger tap).

By neglecting inter-individual BH capacities, we limited the BH time to 4 min for all freedivers for several reasons: (1) we wanted to analyze a comparable amount of stimuli for all participants, for each phase of BH and task in order to avoid strong inter-subject variability in terms of BH times and stimuli presented, which could influence the ERPs; (2) due to the BH ability of 5 min of every freediver, we were certain that all of them would be able to complete the full duration of stimuli presentation; (3) as discussed in greater detail in our previous publication and in “Experimental Tasks: Visual Oddball and Checker Board Reversal Task,” our approach limited the intrusion of muscular artifacts in the EEG due to strong diaphragm contraction during the final phase of pBH as reported previously (Ratmanova et al., 2016).

### Data Processing

After re-referencing EEG data to the arithmetic mean of both ear lobes (A1 and A2), the data were further pre-processed with Butterworth Zero Phase Filters by employing a high-cut-off filter at 50 Hz and low-cut-off filter at 0.5 Hz (24dB/oct). Eye movement corrections were performed by the Gratton and Coles method (Gratton et al., 1983). Lastly, any remaining artifacts were removed utilizing a semiautomatic raw data inspection tool by setting the maximum allowed gradient voltage step at 50 μV/ms, the maximal allowed differences of values in intervals at 100 μV and allowed amplitudes at ±100 μV and visual evaluation. Only segments containing no artifacts were included for further processing.

Electroencephalography recordings during the oddball task were first segmented in time intervals beginning 100 ms before stimulus onset till up to 500 ms after the onset of the target stimulus. Baseline correction to -100–0 ms of stimulus onset was performed. After averaging either across the first 2 min or across the last 2 min, we searched for the latency and the P300 peak amplitude for each electrode in a time frame from 250 to 450 ms using an automatic procedure and visually inspected the peaks. Grand averages across all participants were computed for each block (i.e., first two and last 2 min), each electrode and once for the BH and once for the B condition. The latencies in ms and amplitudes in μV for the two phases (BH and B) were exported for further statistical analysis, but only for Fz, Cz, Pz, and Oz electrodes.

Electroencephalography recordings for the VEPs were also segmented in time intervals from -100 ms of checker reversal onset till up to 300 ms after reversal onset. After correcting for baseline (-100–0 ms of reversal onset), averages were computed separately for the first, second and third VEP phases. After visual inspection of the VEPs, an automatic procedure for detecting the maximal amplitude and the corresponding latency was performed once for the negative N75 (60–120 ms), the positive P100 (121–150 ms) and for the second negative N145 (151–200 ms) component of the VEP. Grand averages were calculated across participants for each VEP phase and for each condition (BH and B), and latencies and amplitudes were exported for the Oz electrode for further statistical analysis. Since O1 and O2 electrodes showed the same VEPs across the BH phases and conditions (see Results section), only Oz statistical analysis is presented here.

Heart rate and Oxygen saturation values were calculated at the beginning of BH after the BH preparation (t0), at one (t1), two (t2), three (t3), and four (t4) minutes by taking the mean value of 5 s before and after the respective points of time. Reaction times to the target stimuli were averaged for each phase and each condition after removing reaction times above 1000 ms and below 100 ms. In summary, ERP data are presented for the oddball task, the first phase being from the start to 2 min (0–2 min) and the second phase being from two to four min (2–4 min). VEP data will be presented according to the three stimuli presentation phases of start to 1 min (VEP-1), 1.5 to 2.5 min (VEP-2) and from 3 min to 4 min (VEP-3).

### Statistical Analysis

Heart rate and Oxygen saturation were each subjected to a 2 × 5 ANOVA with repeated measures on the factor Condition (B and BH) and Time (t0, t1, t2, t3, and t4) separately for the oddball task and the checker board task. For both amplitude and latency of the P300 component elicited by the oddball task, 2 × 2 × 4 ANOVAs were calculated including the within factors Condition (B and BH), Phase (0–2 min and 2–4 min) and Electrode position (Oz, Pz, Cz, and Fz). Reaction time was also subjected to the same ANOVA by excluding the electrode position factor (i.e., 2-way instead of 3-way). Amplitudes and latencies of VEPs over the Oz electrode were analyzed separately for all three components (N75, P100, and N145) by using 2x3 ANOVAs including the within factors Condition (B and BH) and the factor Phase (VEP-1, VEP-2, and VEP-3). In case of significant main effects, Bonferroni-corrected pairwise comparisons were performed to detect the exact loci of significant changes between the factors. Wherever sphericity was violated, Greenhouse-Geisser-adjusted values were reported and p-values below the 5% threshold were considered statistically significant. Effect sizes were estimated according to Cohen (1988) by partial eta-squares (η_p_^2^), where η_p_^2^ > 0.01 indicates a small effect, η_p_^2^ > 0.06 indicates a medium effect and η_p_^2^ > 0.14 indicates a large effect.

## Results

### Oxygenation and Heart Rate Evolution

**FIGURE 2:**
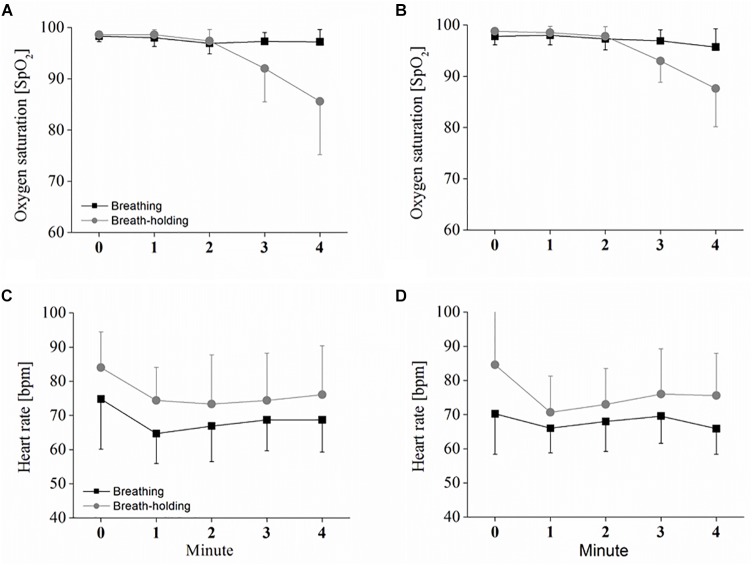
Heart rate (HR) and oxygen saturation in B and BH. Depicted are the oxygen saturation and HR responses to sustained breath-holding and normal breathing, measured once during the oddball task **(A,C)** and obtained once in the checker board task **(B,D)**. Values represent the arithmetic means and error bars represent the corresponding standard deviations. Statistical analysis is presented in the text.

Oxygen saturation evolution over time in the oddball task differed between the two conditions [*F*(1,9) = 6.07; *p* < 0.05; η_p_^2^ = 0.42] and decreased during the BH time [*F*(1.3,11.7) = 6.07; *p* < 0.001; η_p_^2^ = 0.65]. The significant interaction [*F*(1.1,10.3) = 6.07; *p* < 0.01; η_p_^2^ = 0.53] between condition^∗^time revealed that only in the BH condition did a decrease in SpO_2_ occur (see Figure 2A). Bonferroni-corrected pair-wise comparisons further indicate that this decrease started between 2 and 3 min of BH, since SpO_2_ at both 3 and 4 min was significantly lower compared to the start of BH (*p* < 0.05), with SpO_2_ further decreasing between 3 and 4 min of BH (*p* < 0.05). Exactly the same observation was found in the checker board task with significant condition [*F*(1,9) = 5.42; *p* < 0.05; η_p_^2^ = 0.37], time [*F*(1.1,10.0) = 13.01; *p* < 0.01; η_p_^2^ = 0.59] and condition^∗^time interaction [*F*(1.4,13.1) = 16.81; *p* < 0.001; η_p_^2^ = 0.65] effects (see Figure 2B). *Post hoc* comparison again showed that both 3 min and 4 min of BH were significantly lower than at BH start (both *p* < 0.05). However, although a further decrease is visible in the figure, there was no clear, significant difference in SpO_2_ between 3 and 4 min of BH (*p* = 0.10).

As depicted in Figure 2C, HR during the oddball task was also different between the two conditions [*F*(1,9) = 24.68; *p* < 0.001; η_p_^2^ = 0.73] and decreased over the time of B and BH [*F*(2.1,19.4) = 7.98; *p* < 0.01; η_p_^2^ = 0.47]. However, the decrease was not different between B and BH, as indicated by a non-significant interaction effect between condition^∗^time [*F*(1.5,13.6) = 0.28; *p* > 0.05; η_p_^2^ = 0.03]. Pair-wise comparison revealed that the decrease was due to the significant difference between 0 and 1 min (*p* < 0.05). Again, the same HR evolution was found during the checker board task with significant condition [*F*(1,9) = 10.20; *p* < 0.05; η_p_^2^ = 0.56] and time [*F*(1.8,14.6) = 16.76; *p* < 0.01; η_p_^2^ = 0.48] effects, but no significant interaction [*F*(1.7,14.0) = 2.5; *p* > 0.05; η_p_^2^ = 0.24] effect (see Figure 2D). However, Bonferroni-corrected pairwise comparison could detect no clear, significant differences between the single HR measures over time (between 0 and 1 min; *p* = 0.052).

### Oddball Task: P300 and Reaction Time

**FIGURE 3:**
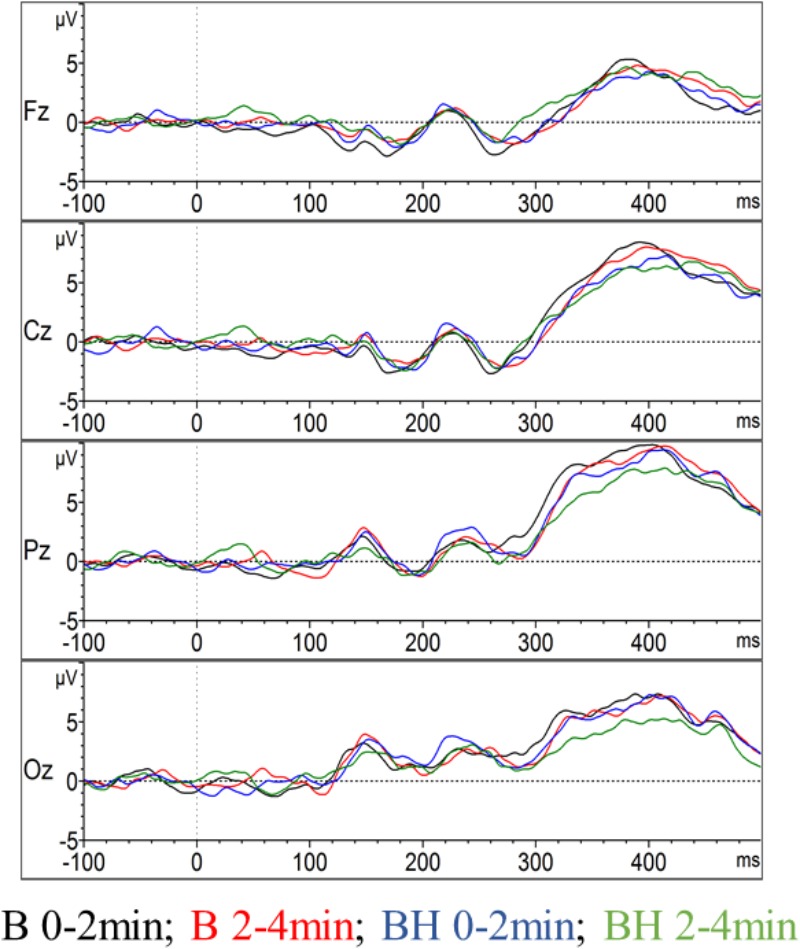
Event-related potentials in the oddball task. The amplitude of the event-related potential P300 evolution for the midline of the brain, including the Oz, Pz, Cz, and Fz electrodes (grand average across all participants). For each electrode position the two conditions with the two phases (0–2 and 2–4 min) are contrasted.

**FIGURE 4:**
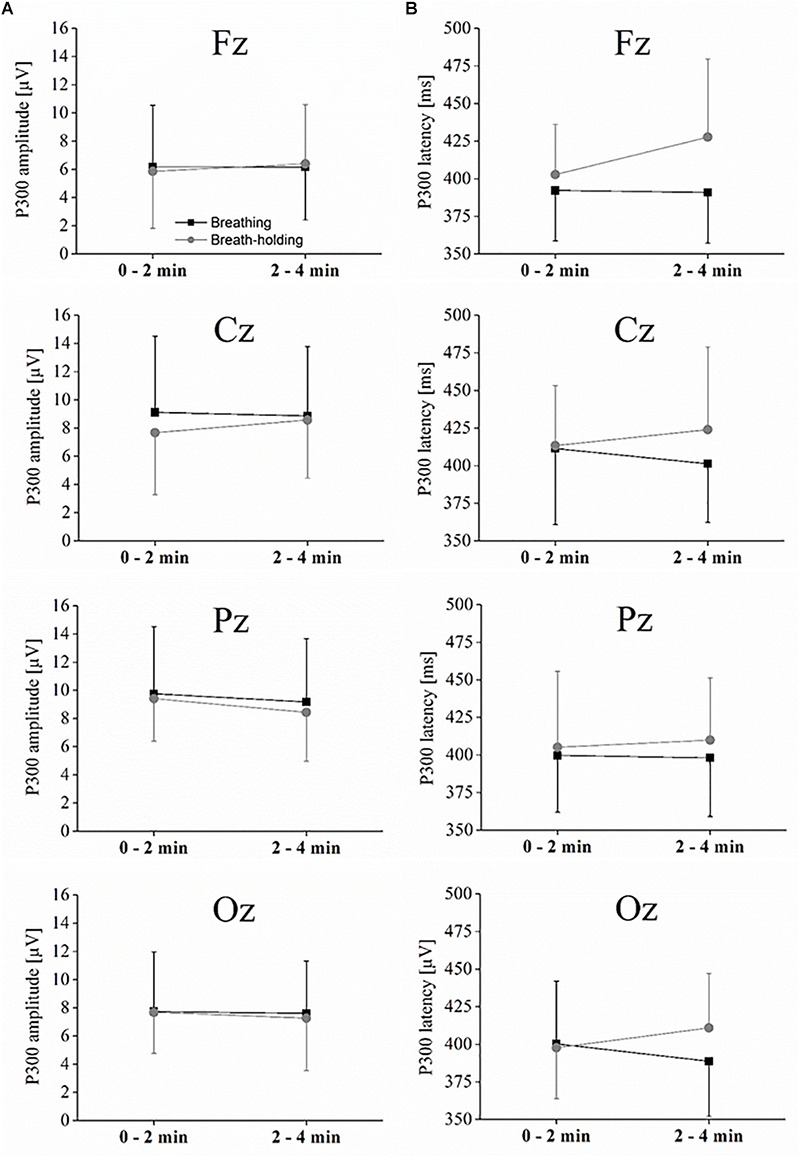
P300 amplitude and latencies. **(A)** Interaction plot showing the peak amplitude for each condition and phase for the midline electrodes. **(B)** Interaction plot showing the latency for each condition and phase for the midline electrodes. Statistics are explained in the text. Values represent the arithmetic means and error bars represent the corresponding standard deviations.

As depicted in Figure 3, the P300 component reached its positive maximum shortly before 400 ms after stimulus onset, which is highest in the Pz electrode position. However, as depicted in Figure 3, 4A, the amplitudes were not systematically different with respect to condition (B or BH) or the phase (0–2 and 2–4 min). This result is confirmed by the 2 × 2 × 4 ANOVA for the amplitude, which detected no significant difference between condition, phase and no significant condition^∗^phase interaction effects (all *p* > 0.05, see Figure 3, 4). As expected, only a significant electrode effect [*F*(1.1,10.3) = 3.6; *p* < 0.05; η_p_^2^ = 0.26] was observed, indicating a higher amplitude for the Pz electrode position. The 2 × 2 × 4 ANOVA including the latencies as the dependent variable revealed a significant condition effect [*F*(1,9) = 7.7; *p* < 0.05; η_p_^2^ = 0.46], indicating overall that latencies were increased in the BH compared to the B condition (see Figure 4B). As visible in Figure 4B, one could assume that latencies, especially in the second BH phase (i.e., 2–4 min), were increased across the whole brain. However, *post hoc* tests did not confirm this visible pattern (all *p* > 0.05), and there were no other main or condition^∗^phase interaction effects of the 2 × 2 × 4 ANOVA (all *p* > 0.05).

**FIGURE 5:**
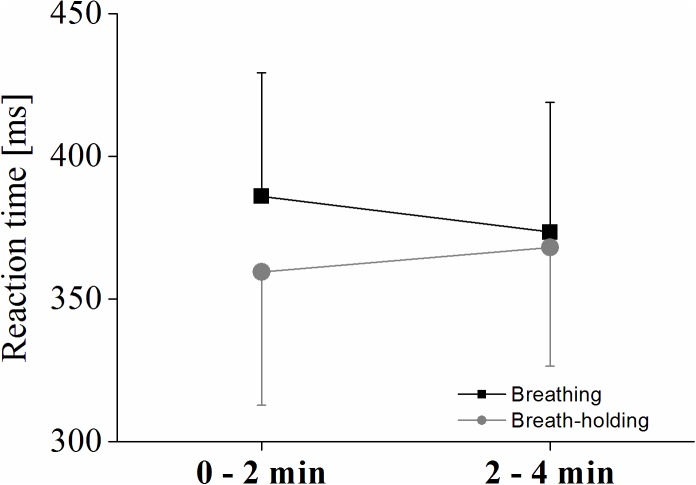
Reaction times for the oddball task. Interaction plot showing the reaction times to target stimuli for each condition and phase. Values represent the arithmetic means and error bars represent the corresponding standard deviations.

A significant condition^∗^phase interaction effect [*F*(1,9) = 8.04; *p* < 0.05; η_p_^2^ = 0.47] of a 2 × 2 ANOVA using participants’ reaction time to the target stimuli shows increased reactions (i.e., decreased performance) in the second phase of the BH, while response time decreased in the normal breathing condition (see Figure 5). However, even though Figure 5 indicates that overall reaction times were slower during normal breathing compared to BH even in the second phase (2–4 min), the condition effect was only significant as a trend [*F*(1,9) = 3.99; *p* = 0.07; η_p_^2^ = 0.30], and no phase effect occurred (*p* > 0.05).

### Checker Board Reversal Task: VEP – N75, P100, and N145

**FIGURE 6:**
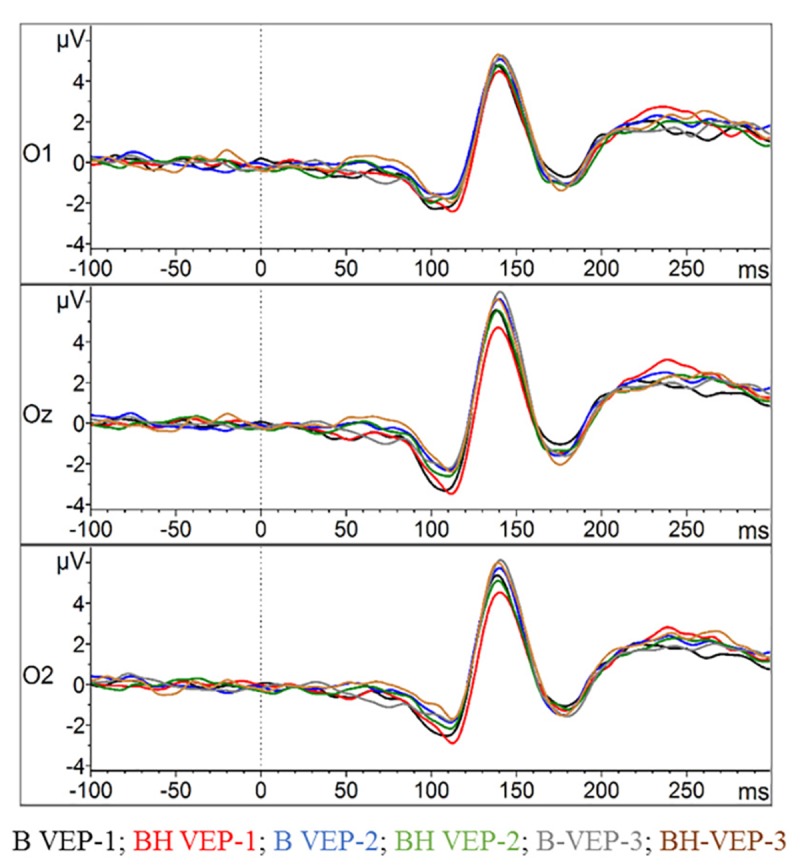
Visual evoked potentials (VEPs) in the checker board task. The amplitudes of the VEPs’ evolution for the occipital lobe at O1, Oz, and O2 (grand average across all participants) are displayed. For each electrode position the two conditions with the three phases of checker board presentation (VEP-1, VEP-2, and VEP-3) are contrasted.

**FIGURE 7:**
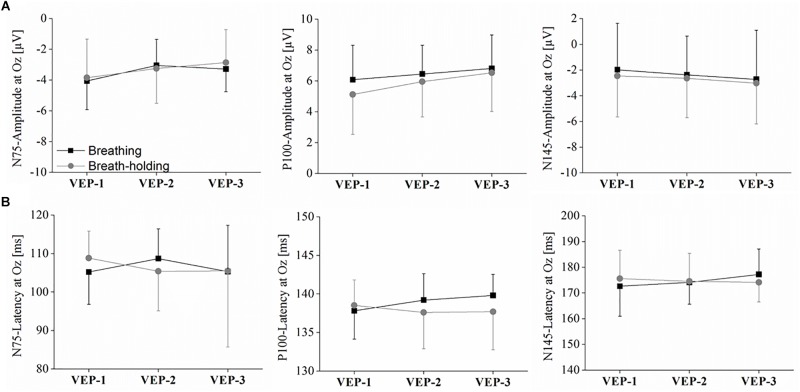
Amplitudes and latencies for the VEPs N75, P100, and N145. **(A)** Interaction plot showing the peak amplitude for each condition and phase for the Oz electrode. **(B)** Interaction plot showing the latency for each condition and phase for the Oz electrode. Statistics are explained in the text. Values represent the arithmetic means and error bars represent the corresponding standard deviations.

As depicted in Figure 6, 7, the VEPs at O1, Oz, and O2 were very similar with respect to condition and phase. Accordingly, no statistical significance was found in the Oz amplitude of any of the three VEP components (N75, P100, and N145) in the 2 × 3 ANOVAs (all *p* > 0.05). The same holds true for the latencies since no strong differences can be detected in the VEP plots (see Figure 6). Consequently, ANOVAs of the latencies in the Oz electrode for N75, P100, and N145 could detect no significant main effect or any condition^∗^phase interactions (all *p* > 0.05).

## Discussion

This experiment recorded for the first time EEG based neuro-cognitive responses in humans to extended breath-holding phases. No significant change either in early visual processing or in neurocognitive markers was measurable during pBH, in trained freedivers. Neither were the amplitudes and latencies of the VEP and the P300 component differently affected between a normal breathing and a breath-holding condition nor was there any difference between an early BH phase and a late BH phase. Thus, the changed oxygen saturation and hypercapnia state elicited by 4 min of BH did not lead to any obvious impairment of the cognitive and visual processing stages.

The results confirm earlier case reports in which Schellart and Reits (1999) performed a pilot study measuring EEG and MEG during pBH of only two subjects, while recording and analyzing VEPs and P300. The results are also in agreement with one EEG-based study which could not detect significant changes in spectral power analysis (Ratmanova et al., 2016). However, they are in contrast to other EEG-based studies that were able to detect changes in neuronal emotional (Menicucci et al., 2014) processing (ERPs) and brain oscillations (Steinberg et al., 2017) due to pBH. The latter study observed changes in the alpha band of the EEG (but no differences in the other frequency bands) and a lateralised modulations in the alpha band (i.e., a stronger left frontal activity indexed by a frontal alpha asymmetry), possibly reflecting the processing of the unique psycho-physiological state, including air-hunger, motivation, self-control and cognitive inhibition (c.f. Steinberg et al., 2017). Thus, these two controversial patterns points toward independent mechanisms: despite apnoea induced modulations in brain oscillation (Steinberg et al., 2017), measured with no competing activity, pBH had no influence in processing of visual information indexed by VEPs or in attention to cognitive tasks, as indexed by the non-affected P300 component in the present approach.

However, the results contradict other hypoxia studies that have consistently found detrimental effects on several cognitive functions for both short and long-term hypoxia (Virués-Ortega et al., 2004, 2006; de Aquino Lemos et al., 2012; Ochi et al., 2018). The results are also in contrast to those hypoxia studies that studied VEP and the P300 component in the EEG, as all such studies have shown that even mild-to-moderate levels of hypoxia modulate the amplitude of VEPs (Forster et al., 1975; Singh et al., 2004) and the latency and the amplitude of the P300 component (Tsarouchas et al., 2008). All those studies attributed the modulations in task-evoked brain activity to the effects of hypoxia, indicating that electrophysiological measures are a sensitive tool to detect detrimental central processing, in the absence of strong behavioral deficits (Tsarouchas et al., 2008). All three studies were performed with a comparable hypoxia level since both the Singh et al. (2004) as well as the Forster et al. (1975) studies were performed in a hypobaric condition comparable with 4300 m altitude and the Tsarouchas et al. (2008) study at an altitude of 4752 m.

However, those studies are not directly comparable to the pBH, as pBH phases include fast and transient changes in blood gas consumptions and respective effects on the brain along different experimental settings (e.g., hypobaric hypoxia). Tests performed in moderate hypoxic conditions were conducted usually after a time delay between the normoxic condition and the hypoxic state (e.g., 5 min in the Tsarouchas et al., 2008 study) or at real altitude, as in the Singh et al. (2004) study. Thus, while SpO_2_ values in these studies were at a lower constant level for a sufficient time to propagate to the brain, they are transient in BH and are also concomitant with transient increases in CO_2_ (Ferretti, 2001). In the later BH phase (2–4min) of the oddball task in the present study, the mean SpO_2_ decreased from 97.4% at BH 2 min to 93.3% at BH 3 min to 85.7% at 4 min. In contrast, in the Tsarouchas et al. (2008) study, the mean SpO_2_ was consistently (i.e., across all task stimuli and thus ERPs not affected by transient levels) lower at 81.52%. Thus, a limitation of our approach is the collapse of stimuli responses after 2 min BH, which might have led to no changes being detectable due to the low hypoxemic state within the brain at this BH phase. It is important to consider, however, that these results of unimpaired brain functions indexed by P300 may only account for experienced freedivers as there is substantial evidence that apnoea training-induced adaptation mechanisms increase oxygen supply to the brain in response to pBH (e.g., Schagatay et al., 2000; Ostrowski et al., 2012; Vestergaard and Larsson, 2017), a mechanism which may not be present in freediving novices or non-divers.

The same argument might also account for the VEP, although the VEP-3 phase was measured between 3 and 4 min of BH; nonetheless, no detrimental effects have been observed, probably also due to the insufficient time available for the hypoxic state to propagate to the occipital lobe of the brain and to provoke any detrimental effects on early visual processing (i.e., mean SpO_2_ at 3 min of BH was 94.8 and 88.5 at 4 min of BH). Singh et al. (2004) found effects on VEPs at high altitude when measured at 4300 m, but that the effects were not considerable at 3200 m. Bearing in mind a review across different hypoxia studies by Virués-Ortega et al. (2004), according to which an altitude of 4700 m corresponds to a mean SpO_2_ of 87%, one might argue that the saturation level beginning from BH 3-min (85.7%) in the Oddball task is comparable to that at a high altitude of about 4700 m, which might in turn provoke hypoxia-related deficits.

However, as pBH-induced blood oxygenation decrease is quite short-lived, it is possible that no strong hypoxemic state can be elicited for up to 4 min of BH that provokes any deficit in brain functions. Since we measured SaO_2_ only at the periphery—a clear limitation of our approach—and due to several brain protecting mechanisms increasing oxygenation supply via modulations of CBF, a firm conclusion cannot be drawn without further physiological measures such as near infrared spectroscopy of the brain or CBF measures (Dujic et al., 2009, 2013; Eichhorn et al., 2015; Bain et al., 2016, 2018a) in combination with longer BH duration (i.e., longer and more constant hypoxemic conditions). Additionally, our results are in contrast to one recent hypercapnia study with MEG and in agreement to another using EEG (Bloch-Salisbury et al., 2000; Thesen et al., 2012). The first measured comparable ERPs during hypercapnia and reported depressed neural activity across the whole cortex and modulated ERPs (Thesen et al., 2012). The latter study could not find any deficit in cognitive processing and in ERPs in hypercapnia (Bloch-Salisbury et al., 2000). Due to these controversial findings along the problematic comparison to the BH-induced hypercapnia state, no firm conclusion can be derived from those findings.

Another interesting finding was the interaction between condition and phase for the response to the oddball stimuli (i.e., the response time for button press when the target stimuli appeared). At the first 2 min phase, reaction times were higher (i.e., slower) in the B condition and lower (i.e., faster) in the BH condition. Then in the second phase of the task (2–4 min), freedivers’ RTs increased in BH and decreased in B, although in both conditions they were comparatively fast. Thus, this pattern points toward different attentional resources allocated to task completion in BH compared to B, and not to hypoxic hypercapnia induced decrease in processing speed. This interpretation agrees with the unaffected P300 latency and amplitude. Therefore, decreased performance in late BH compared to early BH might be attributed to a special kind of dual-tasking situation, since it could well be that attention may be distributed between the processing of all the factors necessary for pBH (c.f. Steinberg et al., 2017) and attention to the visual oddball stimuli, which was not detectable by ERP measures. A dissociation between ERP and associated responses is not a new finding because it is well known from mental chronometry research that changes in RT need not necessarily correlate with the neurophysiological correlate, since the P300 latency does not involve all the factors that contribute to RT (McCarthy and Donchin, 1981). More specifically, the P300 can be described as a kind of real-time index of voluntary attention and requires active information updating in the working memory and the decision making process reflects a “response-related stage” (Polich, 2000; Iv et al., 2010). Thereby, the amplitude of the P300 is an intensity measure of resources that are spent to the allocation of attention (Wickens et al., 1983) and the latency of the P300 is thought to reflect the speed of stimulus classification (Kutas et al., 1977; McCarthy and Donchin, 1981). These distinct processes are thought to be independent of behavioral response times (Verleger, 1997; Ila and Polich, 1999). Therefore, although speculative, those different allocation of attentional resources during the unique BH induced dual-task situation compared to B influence those processing stages that are not reflected in the P300 waveform but in the slowing down of RT in a later BH phase.

As this study was the first with the goal to measure neuro-electrical responses and cognitive processes during BH of extended length, it has some limitations. Two major limitations are of central relevance that need to be considered when interpreting the study. The first is the duration of BH, which was “only” 4 min. This result implied limited changes in blood gas consumption that could have propagated to the brain since stronger changes have been observed in other studies with longer durations of BH up to the individual limit. Second, and interrelated to the first limitation, is the collapsing of data between 2 and 4 min (in the oddball task) or 3–4 min (in the checker board task) as this procedure might have additional blurred any effects in a later BH phase considering the transient nature of blood gas composition during the time course of pBH. Combining these limitations, we cannot yet exclude that brain functions and ERPs are impaired in BH duration at about 4 min or slightly extending 4 min. Further limitations are the lack of additional physiological measures, inclusion of a relatively low number of participants and using a predefined time of BH instead of using individual BH capacity. Thus, our results may not be necessarily transferable to all real freediving activities, especially when they involve significant changes in the hydrostatic pressure level (i.e., deep diving). In turn, our data, results and deduced conclusions, although obtained not during water-immersion, may be most valid for disciplines involving static freediving without any significant pressure changes.

## Conclusion

Despite the fact that pBH requires several unique psycho-physiological processes involving cardiovascular functions that mitigate oxygen supply decreases in sensitive organs, self-control, motivation, and cognitively inhibiting respiratory musculature, no significant changes can be detected in brain electrical responses measured by neuro-cognitive EEG markers (VEP and P300) in experienced free-divers. Whether the lack of changes is due to the capacity of the brain to respond successfully to the hypoxic hypercapnia state during long BH phases or because of limitations in the possibility of reliable measurement of neurocognitive markers at the very end of an apnoea phase needs further investigation in elite apnoea divers, by adding combined physiological and neurophysiological measures along longer BH phases. Given the adaptation mechanisms of freedivers obtained due to training, the assumption of intact brain functions in breath-holding of up to 4 min may only account for the majority of active freedivers, but not for non-divers, freediving novices or elite freedivers performing BHs that regularly extend 4 min or in those disciplines involving changes in the hydrostatic pressure level.

## Ethics Statement

This study was carried out in accordance with the recommendations of Berufsverbandes Deutscher Psychologinnen und Psychologen e.V and the Deutschen Gesellschaft für Psychologie e.V with written informed consent from all subjects. All subjects gave written informed consent in accordance with the Declaration of Helsinki. The protocol was approved by the ethics committee of the ‘Deutsche Gesellschaft für Psychologie.

## Author Contributions

FS wrote the manuscript. MD revised it critically.

## Conflict of Interest Statement

The authors declare that the research was conducted in the absence of any commercial or financial relationships that could be construed as a potential conflict of interest.
